# Disruption of *dmc1* Produces Abnormal Sperm in Medaka (*Oryzias latipes*)

**DOI:** 10.1038/srep30912

**Published:** 2016-08-02

**Authors:** Ji Chen, Xiaojuan Cui, Shaoting Jia, Daji Luo, Mengxi Cao, Yunsheng Zhang, Hongling Hu, Kaiyao Huang, Zuoyan Zhu, Wei Hu

**Affiliations:** 1 State Key Laboratory of Freshwater Ecology and Biotechnology, Institute of Hydrobiology, Chinese Academy of Sciences, No. 7 Donghu South Road, Wuhan 430072, China; 2 University of Chinese Academy of Sciences, Beijing 100049, China; 3 Department of Genetics, School of Basic Medical Sciences, Wuhan University, No. 185 Donghu East Road, Wuhan 430071, China

## Abstract

DMC1 is a recombinase that is essential for meiotic synapsis. Experiments in extensive species of eukaryotes have indicated the independent role of DMC1 in repairing double strand breaks (DSBs) produced during meiosis I. Mutation of *dmc1* in mice and human often leads to obstacles in spermatogenesis and male sterility. Here, we report on the disruption of *dmc1* in male medaka (*Oryzias latipes*). Synapsis was disturbed in the mutant medaka testis nuclei, as observed in mice and other organisms. Unexpectedly, the mutant medaka could produce a few sperm and, although most of these had multiple tail or multiple head malformations, some of them could swim, and few of them even had insemination ability. Our transcriptome analysis showed that there was not a remarkable change in the expression of most of the genes involved in the pathways associated with the meiotic DNA repair and flagella assembly. Our results provided an indication of the accessory mechanisms that might be involved in the repair of DSBs during meiosis. In a species besides humans, we provided evidence that disorders in meiosis recombination might lead to the malformation of sperm.

Infertility is becoming a global threat to human beings. It has been found that 4–17% of couples at childbearing age encounter obstacles in reproduction^1^. One in every twenty males is diagnosed as infertile^2^. Malformation of sperm is thought the most common cause of male infertility^3^,^4^. Sperm are derived from spermatocytes after meiosis and morphological changes. During meiosis, the chromosomes in spermatocytes undergo, in order, homologous pairing, synapsis, recombination and separation. The process of meiosis needs such precise control that errors are prone to occur. It has been estimated that faulty meiosis occurs in 5–25% of human germ cells, which develop into aneuploid gametes; this is thought to be the reason for at least 35% of pregnancy losses and mental retardation cases^5^. Homologous recombination is essential for meiosis; the potential for homologous chromosomes to separate precisely depends on the completion of recombination^6^. A second round of meiosis then occurs, and the gametes are produced. DMC1, a recombinase used in meiosis, plays a vital role during synapsis^7^.

DMC1 is highly conserved in a wide range of eukaryotes^8^. Experiments in mammals indicated that the mutation or deficiency of *dmc1* usually leads to defects in meiotic recombination, accompanied by the accumulation of double strand breaks (DSBs) and abnormal synaptonemal complex formation. For example, the germ cells of *dmc1*^−/−^ mice were arrested at meiosis I and no synaptonemal complexes were observed, then the germ cells underwent apoptosis^9^. Hemizygous mice carrying *mei11*, a dominant negative mutant allele of *dmc1*, were unable to produce sperm cells; the germ cells arrested at pachynema, the structures of the synaptonemal complexes were incomplete, and no crossing over occurred^10^. In human beings, studies of mutants of DMC1 (DMC1-*m200v* and DMC1-*i137*) showed that the mutated proteins were unable to combine the DNA strands steadily under physiological Mg^2+^ or Ca^2+^ conditions, and the patients were sterile^11^,^12^. These reports indicate that DMC1 is essential for the exchange of DNA strands between homologous chromosomes, as well as repairing DSBs during meiosis. DMC1 deficiency causes the abnormal formation of synaptonemal complexes and disordered separation of homologous chromosomes^13^, followed by the apoptosis of germ cells; this leads to the sterility of DMC1 deficient organisms.

Medaka is a small laboratory fish. It is an ideal model for animal development and genetics studies, due to its acclimation to a wide range of living temperatures, small genome size and because it has many mutant strains^14^. Moreover, haploid embryonic stem cell culture and spermatogonial cell culture techniques have been established in medaka, which makes it a good platform to study vertebrate spematogenesis^15^,^16^. Here, we report on the mutation of *dmc1* in male medaka, using transcriptional activator-like effector nucleases (TALENs). In testes of *dmc1*^−/−^ medaka, there were only spermatocytes and a few spermatids. The testes were loose, and there were high rates of cell apoptosis. During meiosis, synapsis of chromosomes in the spermatocytes was disturbed, similar to observations in mammals. Unexpectedly, we found a few sperms formed in the *dmc1*^−/−^ medaka; however, 99% of these had multiple head or multiple tail malformations. These sperm swam slowly and their insemination ability was weak. As far as we know, this is the first report of a vertebrate able to produce sperm with a *dmc1* mutation. Based on the transcriptome data, we surmised that a compensatory pathway existed to repair the DSBs in medaka. Our results indicate that besides the introduction of aneuploidy, disruption to meiosis can also induce malformations in the sperm.

## Results

Using TALENs, we deleted 11 nucleotides in the 3rd exon of the *dmc1* gene, which led to frame shift mutations. The mRNA coded a peptide of 62aa (Fig. S1d). The deduced peptide did not contain any of the functional domains from the original protein (Fig. S2). That was to say that the *dmc1* gene was knocked out.

Compared with the wild type (WT) medaka, there were no differences in growth or reproduction in the *dmc1*^+/−^ hemizygotes. The *dmc1*^−/−^ homozygotes were derived from the hemizygotes by self crossing and showed normal growth. At 4 months post fertilization, when the control medaka reached sexual maturity, the reproductive abilities of the *dmc1*^−/−^ homozygous mutants were tested.

### Synapsis in the *dmc1* ^**−**/**−**^ spermatocytes

**Figure 1:**
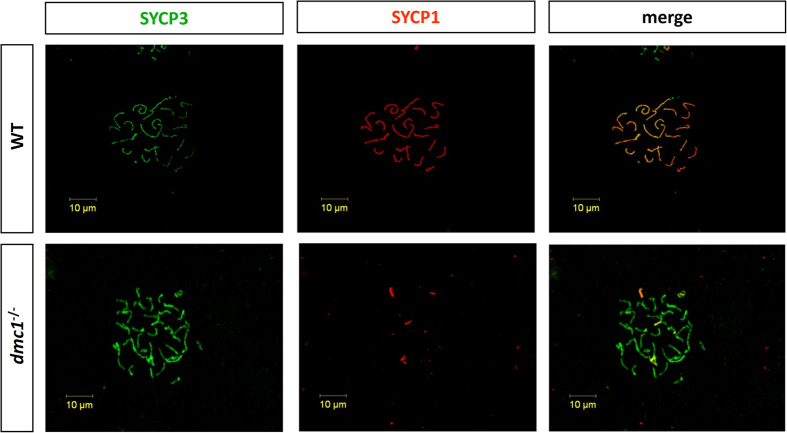
Synapsis and recombination defects in the wild type and mutant testis nuclei.

Using anti-SYCP3 and anti-SYCP1 antibodies, we detected that the SYCP1 protein was unable to assemble to the synaptonemal complex in the spermatocytes of the *dmc1*^−/−^ medaka, indicating defects during synapsis (Fig. 1). The anticipated result also provided evidence that the *dmc1* gene had mutated in the homozygous medaka.

### Apoptosis in the *dmc1* ^−/−^ medaka testes

**Figure 2:**
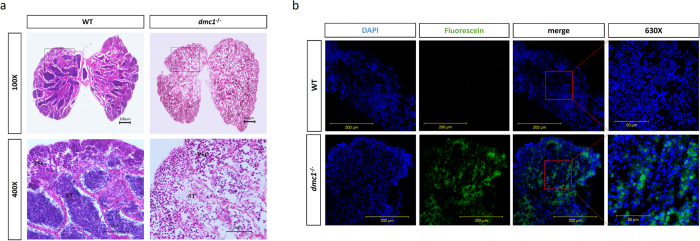
Apoptosis occurred in the *dmc1*^−/ −^ testes. (**a**) Hematoxylin and eosin staining, showing the histology of wild type and mutant testes. (**b**) Apoptosis, detected using terminal deoxynucleotidyl transferase dUTP nick end labeling (TUNEL), in the wild type and mutant testes.

Firstly, we checked the development of the *dmc1*^−/−^ medaka testes using hematoxylin and eosin (H-E) staining; the testes had a loose structure, and the first spermatocytes, with large nuclei, were distributed sparsely. There were many hollow spaces in the seminiferous lobula, with few spermatids. In contrast, the testes of the WT medaka were full of clustered first spermatocytes, second spermatocytes and spermatids (Fig. 2a).

Using terminal deoxynucleotidyl transferase dUTP nick end labeling (TUNEL), we identified the apoptotic cells in the testes of the WT and *dmc1*^−/−^ medaka. In the *dmc1*^−/−^ testes, there were high rates of apoptosis, while nearly no apoptosis occurred in the WT medaka testes (Fig. 2b).

### Malformation in the *dmc1* ^**−**/**−**^ medaka sperm

Since a few spermatids existed in the *dmc1*^−/−^ medaka testes, we analyzed whether they could develop into mature sperm. Firstly, when we pressed the belly of most of the *dmc1*^−/−^ medaka, semen flowed from the cloaca of the fish. Analysis of the semen smear showed mature sperm produced by the *dmc1*^−/−^ medaka; however, their density was lower than that of the WT fish.

**Figure 3:**
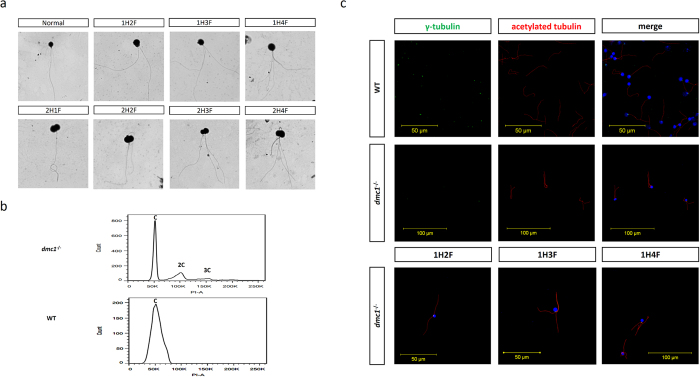
Analysis of the abnormal sperm. (**a**) Morphology of the abnormal sperm, identified by Papanicolaou staining (H: head; F: flagellum). (**b**) Flow cytometry analysis of the *dmc1*^−/−^ and wild type semen. (**c**) Immunofluorescence analysis of the wild type and *dmc1*^−/−^ sperm.

According to standards formulated by WHO^17^, we analyzed the morphology of the sperm. Papanicolaou staining showed that 99.16% ± 0.84% of the *dmc1*^−/−^ medaka sperm had either multiple tail and/or multiple head malformations (Fig. 3a; Table S1); abnormal sperm of one head with multiple tails, two heads with multiple tails, and even three heads with multiple tails were observed. Sperm with one head and two tails accounted for the highest proportion (60%); those with one head and four tails accounted for 7%; two heads and multiple tails accounted for 13%; and those with three heads comprised less than 1% of those present (Fig. S3). Flow cytometry showed that there were haploid, diploid and even triploid sperm in the semen of the *dmc1*^−/−^ medaka, corresponding to those sperm with one, two and three heads, respectively (Fig. 3b). In comparison, there were only 2.23% ± 1.53% malformed sperm in the WT medaka; all of these comprised one head and two tails.

We stained the sperm with acetylated-α-tubulin and γ-tubulin antibodies, which recognize and combine to the flagella and centrosome, respectively^18^. Under the fluorescence microscope, only a pair of centrosomes could be detected in the sperm with one head and multiple tails (Fig. 3c). The observation of multiple flagella from the proximal of one sperm precluded the possibility that the flagella from several sperm overlapped when the semen was dried on the glass slide.

**Figure 4:**
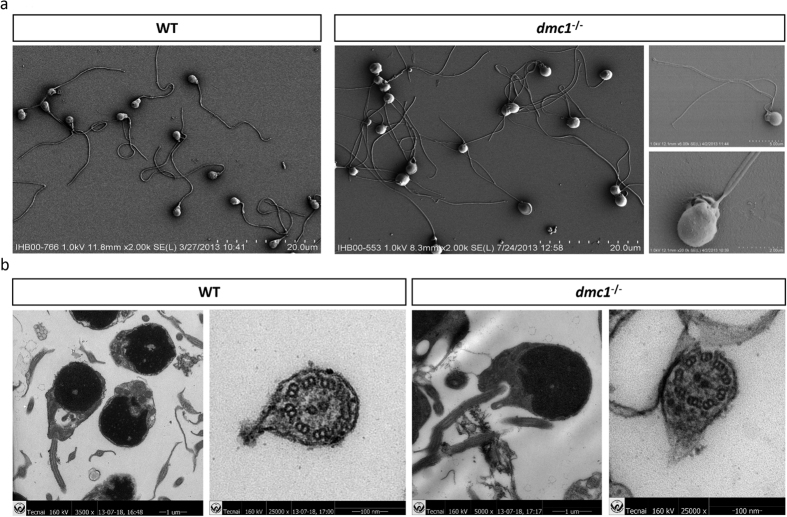
SEM (**a**) and TEM (**b**) analyses of the wild type and *dmc1*^−/−^ sperm.

Further, under SEM, we found that the flagella came from the basal body, instead of branching from a single flagellum (Fig. 4a). The sperm with multiple tails also showed only one pair of centrosomes under the TEM, suggesting that the flagella came from the same centrosomes (Fig. 4b).

Interestingly, among the malformed sperm, there were a few (14.95% ± 13.58%) that could swim irregularly (Supplementary Movie 1). They swam at significantly lower speeds (11.98 ± 4.34 μm/s) and had lower amplitudes of lateral head displacement (0.42 ± 0.19 μm) than the WT sperm (65.53 ± 8.53 μm/s and 1.33 ± 0.20 μm, respectively) (Table S2). The multiple flagella could all flap, with significant lower beat-cross frequencies, but they did have normal microtubule structures (Supplementary Movie 2).

### Insemination ability of the *dmc1* ^−/−^ medaka sperm

To test the insemination ability of the sperm, at the age of sexual maturity (in the wild type sperm), individual male *dmc1*^−/−^ medaka were paired with female WT medaka. The male fish chased the female and provoked the female to lay eggs; all of the eggs died. About 82% (1120/1365) of the eggs were turbid, with the lipid drop having dispersed in the eggs, indicating no fertilization. The rest (245/1365) were transparent, with the lipid drop having gathered at one side; these eggs ceased development after 3–4 cleavages, with 8 or 16 cells arranged irregularly in the animal pole (Fig. S4a).

**Figure 5:**
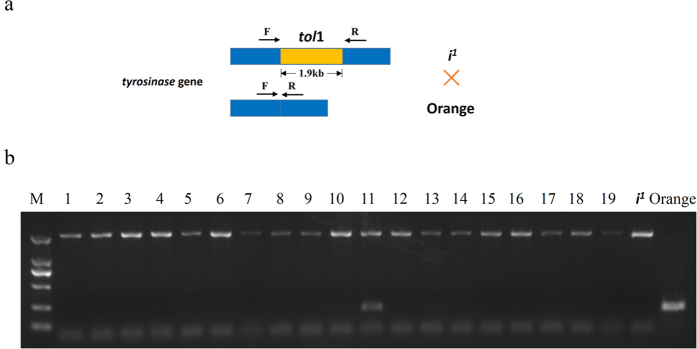
Insemination ability test of the *dmc1*^−/−^ male medaka. (**a**) In the genome of i^1^ strain, there was an insertion of *tol*1 transposon in the *tyrosinase* gene. The *dmc1* mutated medaka was crossed with the i^1^ strain. The genotype of hybrid eggs were checked using PCR primers that flanked the inserted loci. (**b**) The insemination rate was estimated using PCR and agarose electrophoresis.

Judging from their appearance, the eggs seemed to undergo gynogenesis; to confirm whether the eggs were fertilized, we crossed five male *dmc1*^−/−^ medaka with female i^1^ strain medaka. The PCR results illustrated that among the 85 transparent eggs that we tested, there was only one egg in which the genome showed the genotype from both the female and male (Fig. 5).

### Transcriptome analysis of the *dmc1* ^−/−^ testes

**Figure 6:**
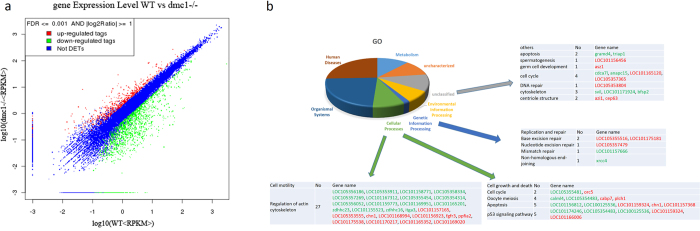
Analysis of the differentially expressed genes between the *dmc1*^−/−^ and wild type testes. (**a**) Dot plot of the differentially expressed genes. (**b**) GO classification of the differentially expressed genes.

From an analysis of the transcriptome, we found that when *dmc1* was mutated, there was a significant change in the expression of 662 genes in the testes; among these genes, 326 were classified into 205 known pathways, including cytoskeleton (27), apoptosis (10), cell cycle (6) and DNA repair (5) pathways. The remaining 336 genes were annotated by their orthologous genes in mammals, and 14 of these genes were associated with cell cycle (4), cytoskeleton (3), centriole structure (2), apoptosis (2), DNA repair (1), spermatogenesis (1) and germ cell development (1) (Fig. 6). We also checked which factors of the orthologous genes in mice were reported to affect spermatogenesis or sperm morphology^19^,^20^,^21^, and meiosis DNA repair pathways (Table S3). Except for the obvious up regulation of *camdm2a* and *camdm2b*, which are associated with the cytoskeleton, there was no significant change in expression of the genes we checked.

Transcriptome raw data are available at the web site: http://www.ncbi.nlm.nih.gov/bioproject/305078.

## Discussion

The process of meiosis is highly conserved in a range of species, from bacteria to humans. Meiosis starts from the induction of DSBs. By repairing the DSBs, genetic information is exchanged between homologous chromosomes^22^,^23^. Meiosis is distinct from mitosis due to the crossing over between homologous chromosomes. As a key recombinase, DMC1 can bind to single stranded DNA to promote the direct exchanges of strands.

Previous research has indicated that, in mammals, spermatocytes with synapsis disorders are eliminated^24^, and that explains the apoptosis of all germ cells in *dmc1* mutated mice^9^. In *dmc1*^−/−^ medaka, however, although synapsis was disturbed, reflected by the defect in synaptonemal complex structure and apoptosis that occurred in the testes, the production of a few sperm confirmed the completion of meiosis in some spermatocytes. The expression of *spo11*, which encodes for the core enzyme that induces strand breaks, did not decrease (Table S3; Fig. S5); this indicates that in the *dmc1*^−/−^ spermatocytes, DSBs as many as in the WT are produced. Most of the *dmc1*^−/−^ spermatocytes underwent apoptosis because the DSBs could not be repaired, similar to observations in mammals. The completion of meiosis in the rest of the spermatocytes suggested that the mechanism for repairing DSBs in medaka is slightly different from that in mammals.

We tried to understand the effect of *dmc1* mutation on meiosis by screening the changes in the expression of genes involved in the regulation of the cell cycle, initiation of meiosis and DNA repair pathways. Unexpectedly, these pathways did not change remarkably, reflected by the stable expression of most of the genes we checked. However, the expression of *xrcc4* gene, which is involved in the non-homologous end-joining (NHEJ) DNA repair pathway, was very significantly reduced (Table S3; Fig. S5). This indicates that the NHEJ pathway is the main way DSBs are repaired in medaka.

RAD51, the core DNA repairase, is prone to mediate the repair processes between sister chromatids in mitosis, and was thought as an accessory factor of DMC1 for mediating the repair between homologous chromosomes. During meiosis in yeast, the expression of *rad51* decreased to reduce competition with *dmc1*, so the DSBs were likely to be repaired using the homologous chromosomes as templates^25^. In *dmc1*^−/−^ spermatocytes, the expression of *rad51* barely changed (Table S3; Fig. S5), indicating that during meiosis *rad51* mainly participated in the NHEJ pathway, instead of the homologous recombination pathway. In fact, there were almost no changes in expression for factors involved in the homologous recombination pathway, such as *rad50*, *rad51d*, *mus81* (Table S3; Fig. S5); this indicates the possible relative independence of the pathways driven by those factors, in which there was no effect on them, even though the NHEJ pathway was blocked.

Through analysis of the transcriptome data, it was clear that several factors (such as LOC105355516, LOC101175181, LOC105357479 and LOC105353804) involved in DNA repair, such as the base excision repair and nucleotide excision repair pathways, were up-regulated (Table S3; Figs 5b and S5). These factors might compensate for the deficiency in repair of DSBs. In fact, a compensatory meiosis mechanism has previously been suspected to exist in human spermatogenesis^26^. The factors above and the not annotated more than 150 factors that were significantly changed in the transcriptome might help to elucidate the mechanism.

Errors during the meiosis of oocytes were the main sources of aneuploidy, rather than defects in the germline precursors^27^. In zebrafish, the mutation of *mlh3*, which encoded for a protein that mediates the repair of DSBs during meiosis, only led to aneuploid gametes, instead of infertility^28^,^29^. In contrast, when DMC1, another protein that mediates the repair of DSBs during meiosis, was disrupted in medaka, not only was meiosis disturbed, but the sperm were also malformed.

Given the observation that more than 97% of the sperm from *dmc1*^−/−^ medaka had multiple flagella, we speculated that something might be wrong with the flagella assembly. Intraflagellar transport (IFT) process is a pathway which mediates protein and vesicle trafficking in primary cilia and flagellum^30^. However, the expression of *ift* in *dmc1*^−/−^ medaka did not change remarkably, in comparison with the WT individuals (Table S3). The TEM results indicate that the flagella of *dmc1*^−/−^ medaka were a microtubule structure of 9 + 2, reflecting a relatively normal structure and assembly of flagella; it is possible that error occurred during the formation of the flagella.

The formation of sperm flagella in *Drosophila* was described by Riparbelli *et al*., indicating the essential role of the accurate assembly, disassembly and migration of centrioles and microtubule for the formation of flagella^31^. In our study, based on the results of the flagella immunofluorescence (acetylated-α-tubulin) and SEM, the multiple flagella likely originate from the basal body, instead of branching off from a single flagellum. The centrioles are the sources of the flagella. Mutation of OAZ or CEP, which are structural proteins in the centrioles, could have caused the abnormal sperms and sterility^32^,^33^. We did not detect remarkable change in expression of *oaz* in the *dmc1*^−/−^ medaka. But the expression of *azi1* and *cep63* did change, suggesting the instability of the centrioles, which could account for the malformation of the sperm (Table S3; Fig. S5).

Several medical cases have suggested that the abnormal isolation of homologous chromosomes in spermatocytes indirectly caused sperm to malform. For example, the mutation of AURKC caused the abnormal isolation of homologous chromosomes, followed by polyploidization, and sperm with big heads and up to six tails^34^. Medical statistical data have indicated that in sterile male patients, there was 2–14% chromosomal aberration. Moreover, defects in sperm structure detected were usually accompanied with DNA fragmentation, fragment loss, immature chromatin or aneuploidy^35^. In our experiment, the mutation of *dmc1* caused disorder in synapsis and the abnormal isolation of homologous chromosomes. Through transcriptome analysis, we surmised that there were changes in the cytoskeleton; the expression of as many as 30 factors involved in the regulation of the actin cytoskeleton pathway was significantly changed, reflecting the abnormal structure of the spermatid cytoskeletons (Fig. 6).

Based on the expression of factors associated with the centrioles and cytoskeleton, we surmised that the mutation of *dmc1* caused abnormal synapsis, followed by uneven isolation of the homologous chromosomes; this, in turn, destabilized the actin cytoskeleton. During the meiosis of the spermatocytes, although the centrioles could move to the anticipated destination, the instability of the cytoskeleton hindered the assembly of the axonemal microtubule, which led to multiple flagella. More experiments are needed to elucidate the pattern and mechanisms involved in this process.

## Materials and Methods

### Ethics Statement

The animals were provided with the best possible care and treatment and were under the care of a specialized technician. Also, all animals were cared for and handled with respect. All procedures were conducted in accordance with the Guiding Principles for the Care and Use of Laboratory Animals and were approved by Institute of Hydrobiology, Chinese Academy of Sciences (Approval ID: keshuizhuan08529).

### Disruption of *dmc1* using TALENs

The TALENs were constructed using the Golden Gate method^36^. The binding-site sequences were 5′-GCA CCG TGA AGG GGA TC-3′ for the left arm, and 5′-GAT GTT GCA CAG AGC CT-3′ for the right arm. The FokI backbone plasmids were PCS2-FokI-KKR and PCS2-FokI-ELD^37^. The constructed plasmids were linearized by NotI (NEB, R0189), and *in vitro* transcribed using an mMESSAGE mMACHINE SP6 kit (Ambion, AM1340), according to the manufacturer’s protocol. Next, the fragments were purified using an RNeasy mini kit (Qiagen, 74104), according to the manufacturer’s protocol. The resultant mRNA for the left and right arms was mixed in equal proportions and diluted to 300 ng/μL (total mRNA). The RNA mixture (2 nL) was microinjected into one-cell stage embryos of the orange strain; DNase/RNase free water was used as a negative control.

The fish were maintained under artificial conditions with a 14/10 h light/dark cycle, at 26.5 °C. The P0 fish were crossed with the WT fish to produce F1 embryos. Genotypes were screened by PCR, using randomly selected embryos (Fig. S1). The △ 11 fish were test crossed to get F2 hemizygotes. The homozygotes used were from the F2 generation, achieved by self crossing.

### Papanicolaou staining

The semen was diluted by Hank’s solution before being smeared to the polylysine treated glass slides. After air-drying, the specimens were stained with Mayer’s hematoxylin, G6 and EA50 dyes, in order.

### Flow cytometry

The semen was washed with Hank’s solution three times, and fixed using 70% ethanol at −20 °C overnight. Then, the ethanol was removed and the sperm were suspended using Hank’s solution with 10 μg/mL RNase and 50 μg/mL propidium iodide. After filtration through a 40 μm cell strainer, the suspension was analyzed using a FACSAria III flow cytometer (BD).

### Immunofluorescence of sperm and synaptonemal complexes

The sperm specimens were permeabilized using Triton X-100. Then they were incubated at 4 °C overnight with anti-acetylated-α-tubulin and anti-γ-tubulin antibodies at dilutions of 1:1000 and 1:200, respectively. The second antibodies were Dylight488 anti-rabbit IgG and Dylight549 anti-mouse IgG. Images were captured under a Zeiss LSM710 cofocal microscope.

SYCP3 was a component of the axial/lateral synaptonemal complex elements and SYCP1 was a component of the synaptonemal complex transverse filaments, which mark the synapsed chromosome regions^38^,^39^. According to Iwai *et al*.40, we expressed the SYCP3 and SYCP1 proteins of medaka in *E. coli* and obtained polyclonal anti serum from rabbits and mice, respectively. Bivalents of medaka spermatocytes were prepared as described by Kochakpour^41^. Immunofluorescence was carried out using the polyclonal serum.

### SEM and TEM analyzes

The sperm specimens were fixed using 2.5% glutaraldehyde, then dropped onto a round glass slide and dried using a silica gel drier. After the surface was treated with electric conduction, the specimens were observed under a Hitachi S-4 scanning electron microscope.

The fixed sperm specimens were centrifuged into a block and cut into an ultramicrocut. After the surface was treated with electric conduction, the specimens were observed under a Hitachi H-7700 transmission electron microscope.

### Vitality test

The semen was diluted by 1% BSA, and immediately observed under an Olympus IX51 microscope. Parameters of sperm vitality (curvilinear velocity, straight-line velocity, average path velocity, amplitude of lateral head displacement, beat-cross frequency.) were measured using a software made by Yangtze River Fisheries Research Institute, Chinese Academy of Fishery Sciences.

### Insemination ability test

Eight pairs of WT medaka were selected for crossing (one to one). The numbers of eggs produced and those that survived were recorded. Every 5–7 days, the pairing would be rotated and survival rates would again be recorded. After three rounds of rotation, the female medaka with good fertilization abilities were selected to be paired with male *dmc1*^−/−^ medaka (Table S4).

Female adult medaka from the i^1^ strain^42^ were used to test cross with the *dmc1*^−/−^ male adults. The transparent eggs after 3 hpf were collected. Genotypes of the transparent eggs were screened using PCR.

### Transcriptome analysis

RNA was extracted from the intact testes of adult *dmc1*^−/−^ and WT (4 month old) specimens. Then, their cDNA libraries were constructed. A transcriptome (2G) was obtained by sequencing the cDNA libraries, using the Illumina Hiseq2000 sequencer. The transcriptome was annotated using the medaka genome database. The P-value was corrected by Bonferroni through Fisher’s exact test^43^. Those genes with changes in expression of more than 2 fold, FDR values of less than 0.001 and RPKM values of more than 4 were considered to be confidently significantly differentially expressed genes. These genes were classified by GO (Gene Ontology) and KEGG (Kyoto Encyclopedia of Genes and Genomes) pathway analysis. Unclassified genes were annotated by referring to their orthologous genes in mammals.

For some differentially expressed genes and the concerned genes whose FDR values were higher than 0.001, primers were designed (Table S5), and real-time PCR was performed on a Roche 480II machine, to test the data reliability (Fig. S5).

## Additional Information

**How to cite this article**: Chen, J. *et al*. Disruption of *dmc1* Produces Abnormal Sperm in Medaka (*Oryzias latipes*). *Sci. Rep*. **6**, 30912; doi: 10.1038/srep30912 (2016).

## Supplementary Material

Supplementary Information

Supplementary Movie S1

Supplementary Movie S2
