# Supplementary material for: Disruption of dmc1 Produces Abnormal Sperm in Medaka (Oryzias latipes)
Source: Sci Rep. 2016 Aug 2;6:30912. doi: 10.1038/srep30912 (PMC4969596; doi:10.1038/srep30912)
Supplement: Supplementary Information [file srep30912-s1.doc]

**Disruption of *dmc1* Produces Abnormal Sperm in Medaka (*Oryzias latipes*)**

Ji Chen^1,*^, Xiaojuan Cui^1,*^, Shaoting Jia^1,2^, Daji Luo^3^, Mengxi Cao^1^, Yunsheng Zhang^1^, Hongling Hu^1,2^, Kaiyao Huang^1^, Zuoyan Zhu^1^, Wei Hu^1^

^1^State Key Laboratory of Freshwater Ecology and Biotechnology, Institute of Hydrobiology, Chinese Academy of Sciences, No. 7 Donghu South Road, Wuhan 430072, China. ^2^ University of Chinese Academy of Sciences, Beijing 100049, China. ^3^ Department of Genetics, School of Basic Medical Sciences, Wuhan University, No. 185 Donghu East Road, Wuhan 430071, China.

^*^These authors contributed equally to this work.

Correspondence and requests for materials should be addressed to Wei Hu (email: huwei@ihb.ac.cn).

**Supplementary files**

**Supplementary Table S1. Malformation rates of the *dmc1*^−/−^ sperm.**

|  | Numbers of sperms | Numbers of normal sperms | Numbers of malformed sperms | Malformation rates（%） |
| --- | --- | --- | --- | --- |
| wt-1 | 985 | 948 | 37 | 3.76 |
| wt-2 | 1947 | 1899 | 48 | 2.47 |
| wt-3 | 1229 | 1208 | 21 | 1.71 |
| wt-4 | 1239 | 1198 | 41 | 3.31 |
| wt-5 | 1658 | 1638 | 20 | 1.21 |
| wt-6 | 996 | 989 | 7 | 0.7 |
| KO-1 | 830 | 14 | 816 | 98.31 |
| KO-2 | 577 | 0 | 577 | 100 |
| KO-3 | 985 | 1 | 984 | 99.9 |

**Supplementary** **Table S2. Vitality measurements of the wild type and *dmc1*^−/−^ sperms.** Grade A: fast forward movement; Grade B, slow forward movement; Grade C, no forward movement; Grade D, no movement; VCL, curvilinear velocity; VSL, straight-line velocity; VAP, average path velocity; ALH, amplitude of lateral head displacement; BCF, beat-cross frequency.

|  | Grade A(%) | Grade B(%) | Grade C(%) | Grade D(%) | VCL(um/s) | VSL(um/s) | VAP(um/s) | ALH(um) | BCF(times/s) |
| --- | --- | --- | --- | --- | --- | --- | --- | --- | --- |
| WT  **n=16** | 90.44±5.52 | 4.87±1.56 | 0.46±0.38 | 4.23±4.93 | 76.3±9.70 | 52.57±7.17 | 65.53±8.53 | 1.33±0.20 | 4.31±0.19 |
| KO  **n=9** | 6.09±5.93** | 6.94±8.07** | 0.55±0.95 | 86.64±13.49 ** | 15.91±5.66 ** | 10.49±3.95 ** | 11.98±4.34 ** | 0.42±0.19 ** | 2.5±0.67 ** |

**Supplementary** **Table S3. Changes in the expression genes analyzed.**

| Gene | Full name | Pathway | Fold change (log2） | RPKM in MWTMA | RPKM in MKOMA | FDR |
| --- | --- | --- | --- | --- | --- | --- |
| mus81 | crossover junction endonuclease MUS81 | HRR & Fanconi anemia pathway | -0.23488 | 16.93153 | 14.38767 | 0.000256 |
| ruvbl2 | RuvB-like 2 | Homologous recombination | -0.15879 | 45.26093 | 40.54373 | 0.000258 |
| rad51d | DNA repair and recombination protein RAD51d | Homologous recombination | 0.254925 | 21.58631 | 25.75837 | 2.40E-06 |
| rad51 | DNA repair protein RAD51 | Homologous recombination | -0.1981 | 27.48967 | 23.96272 | 0.003519 |
| smc5 | structural maintenance of chromosomes protein 5 | Homologous recombination | -0.58729 | 35.93657 | 23.91904 | 5.30E-62 |
| smc6 | structural maintenance of chromosomes protein 6 | Homologous recombination | -0.19548 | 108.4412 | 94.69985 | 1.60E-27 |
| rint1 | RAD50 interactor 1 | HRR & NHEJ | 0.457843 | 6.842301 | 9.397808 | 2.37E-08 |
| xrcc4 | non-homologous end-joining DNA-repair protein XRCC4 | Non-homologous end-joining DNA-repair | -3.03318 | 4.363774 | 0.533069 | 1.96E-61 |
| rad50 | double strand break repair protein RAD50 | DNA double-strand break repair | 0.079272 | 47.18463 | 49.84986 | 0.002383 |
| smc1a | meiosis-specific cohesin subunit SMC1 alpha | meiosis | -0.1616 | 22.18348 | 19.83283 | 2.19E-05 |
| mns1 | meiosis-specific nuclear structural protein 1 | meiosis | 0.116509 | 190.9131 | 206.9705 | 8.51E-10 |
| smek2 | SMEK homolog 2, suppressor of mek1 | DNA replication | 0.187248 | 90.08008 | 102.5643 | 1.65E-22 |
| rpa1 | replication protein A1, 70kDa | DNA replication | -0.10743 | 55.88744 | 51.8771 | 0.00271 |
| topbp1 | topoisomerase (DNA) II binding protein 1 | DNA replication | --0.14932 | 14.77538 | 13.32261 | 0.001462 |
| atrX | transcriptional regulator ATRX-like | transcription regulation | -0.07098 | 51.33274 | 48.8682 | 0.000164 |
| ube2h | ubiquitin-conjugating enzyme E2H | transcription regulation | 0.158382 | 61.66281 | 68.81781 | 0.000196 |
| cd3eap | CD3e molecule, epsilon associated protein | transcription regulation | -0.19627 | 79.45207 | 69.34597 | 1.22E-06 |
| taf4 | transcription initiation factor TFIID subunit 4 | transcription regulation | -0.20744 | 16.20072 | 14.03104 | 0.00018 |
| ift52 | intraflagellar transport protein 52 homolog | flagellar assembly | -0.3584 | 149.008 | 116.2305 | 7.35E-48 |
| ift57 | intraflagellar transport protein 57 homolog | flagellar assembly | -0.15159 | 27.48115 | 24.74013 | 9.22E-05 |
| ift74 | intraflagellar transport protein 74 homolog | flagellar assembly | 0.29244 | 36.50392 | 44.70672 | 3.34E-08 |
| ift81 | intraflagellar transport protein 81 homolog | flagellar assembly | 0.141936 | 106.3094 | 117.3002 | 1.22E-16 |
| ift88 | intraflagellar transport protein 88 homolog | flagellar assembly | 0.397418 | 50.22279 | 66.15089 | 2.17E-55 |
| ift122 | intraflagellar transport protein 122 homolog | flagellar assembly | 0.379242 | 65.31417 | 84.95139 | 2.03E-81 |
| ift140 | intraflagellar transport protein 140 homolog | flagellar assembly | -0.30365 | 37.75506 | 30.58924 | 5.69E-44 |
| ift172 | intraflagellar transport protein 172 homolog | flagellar assembly | -0.07482 | 119.1761 | 113.1531 | 3.18E-07 |
| znf423 | ornithine decarboxylase antizyme | centrosomal protein | 0.349484 | 6.84835 | 8.725514 | 4.72E-09 |
| mad2l1 | MAD2 mitotic arrest deficient-like 1 | mitotic spindle assembly checkpoint | 0.30678 | 10.2478 | 12.67595 | 0.000669 |
| bub1b | BUB1 mitotic checkpoint serine/threonine kinase B | spindle structure | 0.769989 | 47.18954 | 80.4703 | 5.33E-123 |
| anapc10 | anaphase-promoting complex subunit 10 | cell cycle | 0.925369 | 50.14007 | 95.22453 | 7.48E-96 |
| tex11 | testis expressed gene 11 | spermatogenesis | -0.13878 | 51.4422 | 46.72417 | 2.27E-09 |
| LOC105355516 | von Willebrand factor A domain-containing protein 5A-like | DNA repair | 1.344663 | 5.21311 | 13.23978 | 3.75E-71 |
| LOC101175181 | von Willebrand factor A domain-containing protein 5A-like | DNA repair | 4.360863 | 14.67117 | 1.750298 | 1.35E-31 |
| LOC105357479 | uncharacterized | DNA repair | 2.280474 | 7.269194 | 1.672462 | 1.63E-90 |
| LOC105353804 | uncharacterized | DNA repair | 2.835515 | 9.841273 | 1.795234 | 1.16E-11 |
| sfr1 | SWI5-dependent recombination repair 1 | DNA repair | 0.355231 |  |  | ＞0.001* |
| nsmce4a | non-SMC element 4 homolog A | DNA repair | 0.352671 |  |  | ＞0.001* |
| hormad1 | HORMA domain-containing protein 1 | DNA double-strand break repair | 0.220092 |  |  | ＞0.001* |
| msh6 | DNA mismatch repair protein MSH6 | Mismatch repair | 0.927547 |  |  | ＞0.001* |
| spo11 | meiotic protein covalently bound to DSB homolog | meiosis | 0.419999 |  |  | ＞0.001* |
| smc1b | meiosis-specific cohesin subunit SMC1 beta | meiosis | 0.195994 |  |  | ＞0.001* |
| mei4 | meiosis-specific protein MEI4-like | meiosis | 0.796102 |  |  | ＞0.001* |
| rpa2 | replication protein A 32 kDa subunit | DNA replication | 0.871543 |  |  | ＞0.001* |
| azi1 | 5-azacytidine induced 1 | centrosomal protein | 2.033688 |  |  | ＞0.001* |
| cep63 | centrosomal protein 63 | centrosomal protein | 1.323261 |  |  | ＞0.001* |
| fbxo5 | F-box protein 5 | Early mitotic inhibitors | 0.357818 |  |  | ＞0.001* |
| taz | tafazzin | spermatogenesis | 0.102839 |  |  | ＞0.001* |
| hook1 | hook microtubule-tethering protein 1 | spermatogenesis | 0.788048 |  |  | ＞0.001* |

* For the genes whose FDR value were higher than 0.001, the expression changes were verified by realtime-PCR.

**Supplementary** **Table S4. Fertility tests of the *dmc1*^−/−^ male medaka.** (A) Cross of wild type females and males. (B) Cross of the same wild type females and *dmc1*^−/−^ males.

A

|  | WT-1 | WT-2 | WT-3 | WT-4 | WT-5 | WT-6 | WT-7 | WT-8 |
| --- | --- | --- | --- | --- | --- | --- | --- | --- |
| 1st round | 4/12 | 20/22 | 9/13 | 30/34 | 20/20 | 16/16 | 28/32 | 15/16 |
| 2nd round | 75/85 | 34/39 | 19/24 | 31/35 | 71/77 | 76/89 | 12/12 | 24/38 |
| 3rd round | 8/10 | 23/29 | 23/33 | 6/6 | 24/31 | 27/27 | 3/6 | 38/71 |

B

|  | KO-1 | KO-2 | KO-3 | KO-4 | KO-5 | KO-6 | KO-7 | KO-8 |
| --- | --- | --- | --- | --- | --- | --- | --- | --- |
| 1st round | 0/46 | 0/13 | 0/46 | 0/35 | 0/21 | 0/22 | 0/26 | 0/47 |
| 2nd round | 0/15 | 0/37 | 0/50 | 0/44 | 0/57 | 0/56 | 0/85 | 0/25 |
| 3rd round | 0/63 | 0/21 | 0/36 | 0/20 | 0/39 | 0/50 | 0/18 | 0/16 |
| 4th round | 0/56 | 0/26 | 0/43 | 0/26 | 0/23 | 0/40 | 0/10 | 0/36 |
| 5th round | 0/32 | 0/49 | 0/18 | 0/28 | 0/43 | 0/9 | 0/24 | 0/24 |

**Supplementary Table S5. Genes and primers list in quantitative analyses.**

| Detector | Primer Name | Primer sequence |
| --- | --- | --- |
| anapc10 | anapc10-F | 5'-CAACCAgCgGACCAACG-3' |
|  | anapc10-R | 5'-CACCggCgTgTAgACCTT-3' |
| azi1 | azil-F | 5'-CTggTCCgCCTgCAgACTTT-3' |
|  | azil-R | 5'-CgCTCTgCAAACTCCTgCCT-3' |
| camdm2a | camdm2a-F | 5'-ACggAgTTgAACTTCCAgACC-3' |
|  | camdm2a-R | 5'-gTTgAgCgAAgTgAAggTgAg-3' |
| camdm2b | camdm2b-F | 5'-CACAgCCAAgAATgAAAAgg-3' |
|  | camdm2b-R | 5'-AgCCAgATACCgTCCAACA-3' |
| cep63 | cep63-F | 5'-AgAgAgCAgCACTgCAgCCA-3' |
|  | cep63-R | 5'-CAggACTTgATgACgggATTgA-3' |
| fbxo5 | fbxo5-F | 5'-gTgTCgCTgCTgTCCAggAA-3' |
|  | fbxo5-R | 5'-CTggCACCTggTgTgAgCTg-3' |
| hook1 | hook1-F | 5'-gAATCCTgCCACCgCTgAgA-3' |
|  | hook1-R | 5'-ACCAggCggTgACgATTAgC-3' |
| hormad1 | hormad1-F | 5'-ggAggCgAACCTgAAgTggA-3' |
|  | hormad1-R | 5'-TTTgggCTCgCTgAATTTgC-3' |
| LOC101175181 | LOC101175181-F | 5'-TggTTTATgCCCAACTCTgTg-3' |
|  | LOC101175181-R | 5'-CgAATCAAAATACgAgCAgCTA-3' |
| LOC105353804 | LOC105353804-F | 5'-AgTggATTgCCAACTCCTCTC-3' |
|  | LOC105353804-R | 5'-gCTTCAACTCATCTTAgTTAggCA-3' |
| LOC105355516 | LOC105355516-F | 5'-TCTTCAgTgACTCCTggTCgT-3' |
|  | LOC105355516-R | 5'-ACTCTACTTTggAAACTggACg-3' |
| LOC105357479 | LOC105357479-F | 5'-CAGAGGAGACGCAGCGAGGT-3' |
|  | LOC105357479-R | 5'-GGAGGAGCGACAATCCTTGG-3' |
| mei4 | mei4-F | 5'-AgTCgCATggAggAgCCACT-3' |
|  | mei4-R | 5'-TCCATCAgCCTCTCTgCATCC-3' |
| mnd1 | mnd1-F | 5'-CAACTACTACTgggCgTTTCC-3' |
|  | mnd1-R | 5'-gTCgCTTTCCgCTgCTTA-3' |
| msh6 | msh6-F | 5'-gggTTCAACgCTgCTCgACT-3' |
|  | msh6-R | 5'-ACAACCggAggCTgACggTA-3' |
| mus81 | mus81-F | 5'-gCgAgCAAAAgTTTCgTCTA-3' |
|  | mus81-R | 5'-CACCACCTgCgTgTTCACTA-3' |
| nsmce4a | nsmce4a-F | 5'-ggAAgCCAAACggATCATgC-3' |
|  | nsmce4a-R | 5'-ggAgATgTCgggTCgTCTTgA-3' |
| oaz | oaz1-F | 5'-TTCCTgAAggCAgCAAggAgA-3' |
|  | oaz1-R | 5'-CgCACCAgTTTAgCACgATCA-3' |
| rad50 | rad50-F | 5'-gTggATgAAAATgCCTCTgC-3' |
|  | rad50-R | 5'-AggCTAAAACCTTCTgTCCg-3' |
| rad51 | rad51-F | 5'-CCgTgATCgTggACTCTgTg-3' |
|  | rad51-R | 5'-gAAgTCCTTTgCCATCgTCT-3' |
| rad51d | rad51d-F | 5'-gggAggCAAgCAAAATgAAg-3' |
|  | rad51d-R | 5'-TggTCACCAgAACAgCAACg-3' |
| rad54 | rad54-F | 5'-TCACTgggCgAgCTACgAg-3' |
|  | rad54-R | 5'-gACCTgCCTgCCATTCACA-3' |
| rpa2 | rap2-F | 5'-gACAggTgCAggAATgCTCAgA-3' |
|  | rap2-R | 5'-CTTCTgCCCTggACCTCTgC-3' |
| sfr1 | sfr1-F | 5'-gCTCCCAgAACgCgCTgTAT-3' |
|  | sfr1-R | 5'-gggCggTCAAAgTgCAgAAA-3' |
| smc1b | smc1b-F | 5'-CgCAgCTCTCgCCTTTCTgT-3' |
|  | smc1b-R | 5'-gTCAAgAgCCgCATCCACCT-3' |
| spo11 | spo11-F | 5'-gCAgggCTTTgCATgTgATg-3' |
|  | spo11-R | 5'-TgCCggAgACATTTgACgAA-3' |
| taz | taz-F | 5'-TgCTTCACCAAAgAgCTgCAC-3' |
|  | taz-R | 5'-TgTgCACCCATTCACCTCTg-3' |
| xrcc4 | xrcc4-F | 5'-AACACgATACATTTgggCTCT-3' |
|  | xrcc4-R | 5'-CTCAgTCTgTggTTCTCTgCTAA-3' |

**
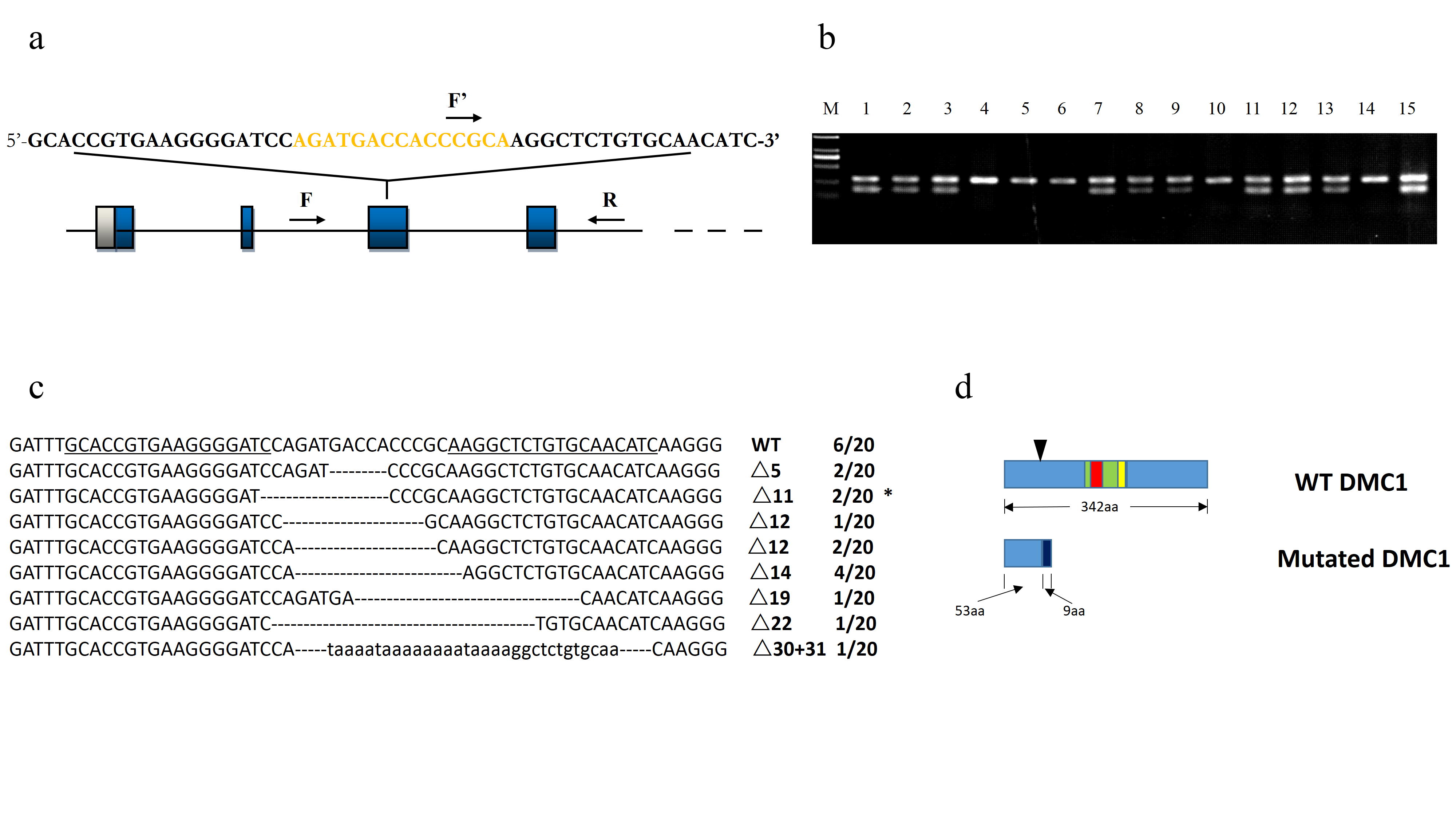
**

**Supplementary** **Figure S1** The targeted mutation of *dmc1* using transcriptional activator-like effector nucleases (TALENs)**.** (a) The target was designed on the 3rd exon of the *dmc1* gene. (b) Screening of the mutated DNA fragments using tri-primer PCR and agarose electrophoresis. (d) Verification of the genotype by sequencing; *the △11 genotype was used to test cross. (d) The deduced protein of the mutant gene.


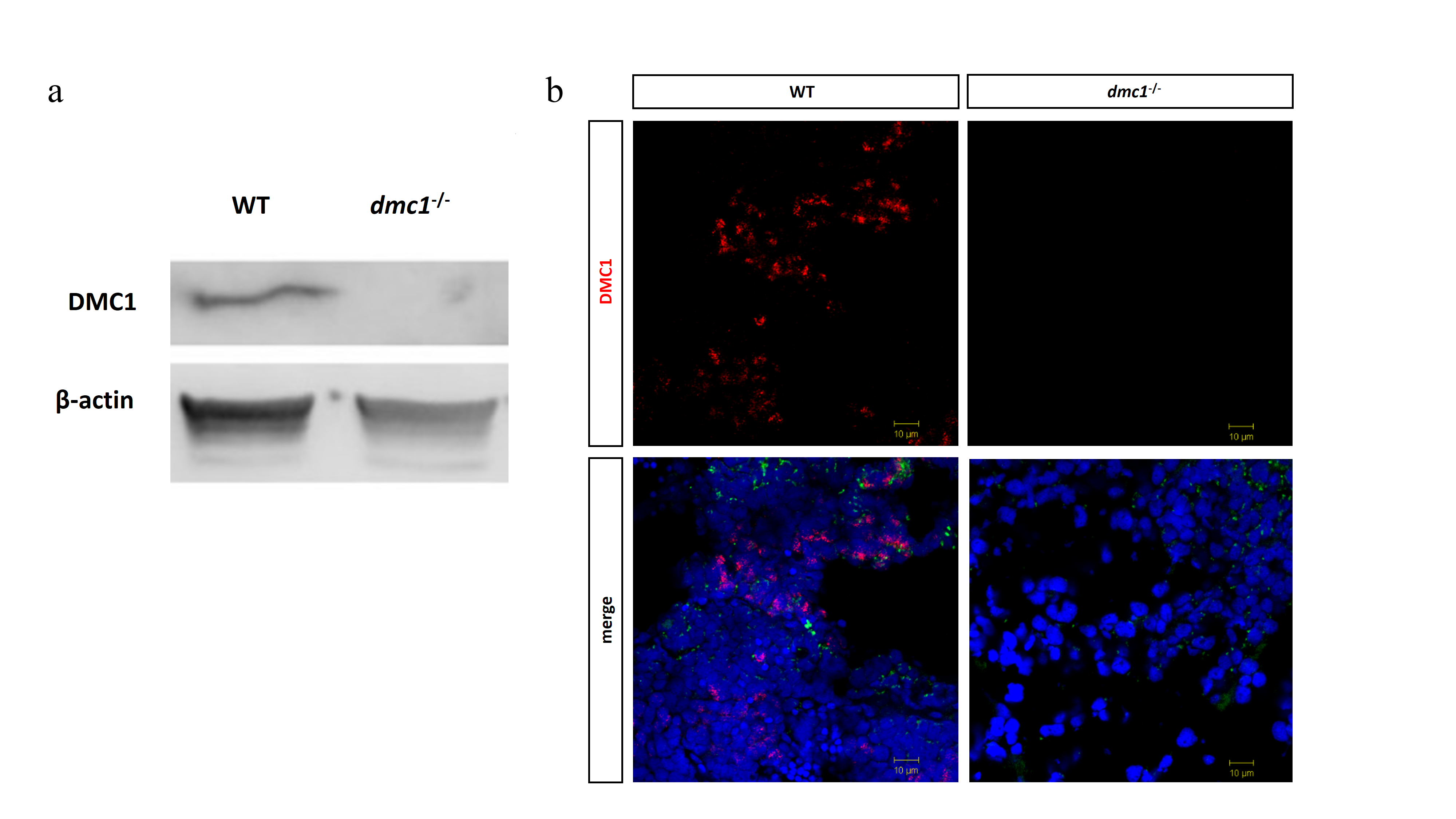


**Supplementary** **Figure S2** Confirmation of DMC1 deficiency in the mutant testis. (a) Western blot analysis. (b) Immunofluorescence of the germline markers VASA and DMC1 in the testis (blue: DAPI; green: VASA; red: DMC1).


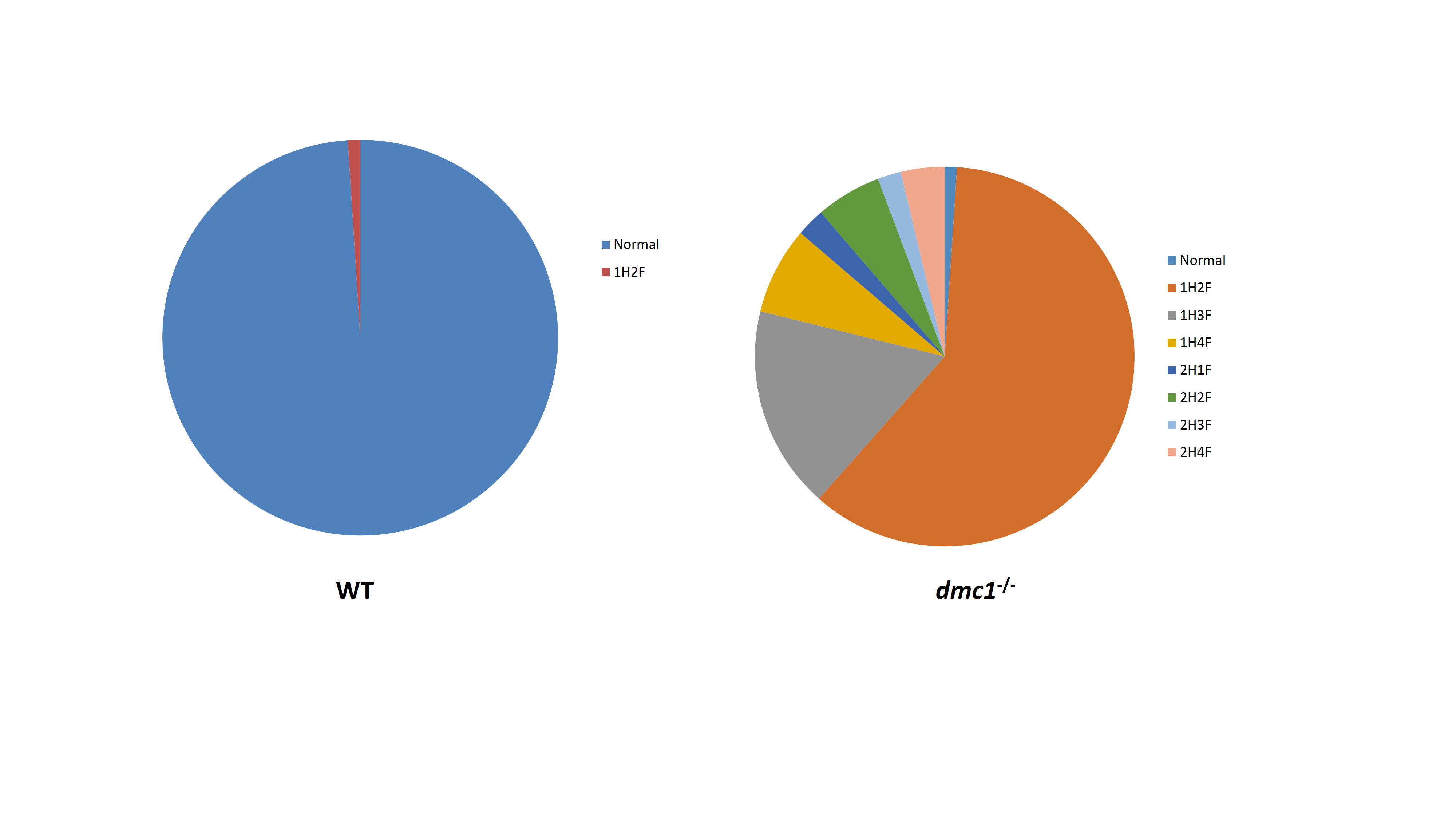


**Supplementary** **Figure S3** Descriptive statistics of the types of malformed sperm. H: head; F: flagella.


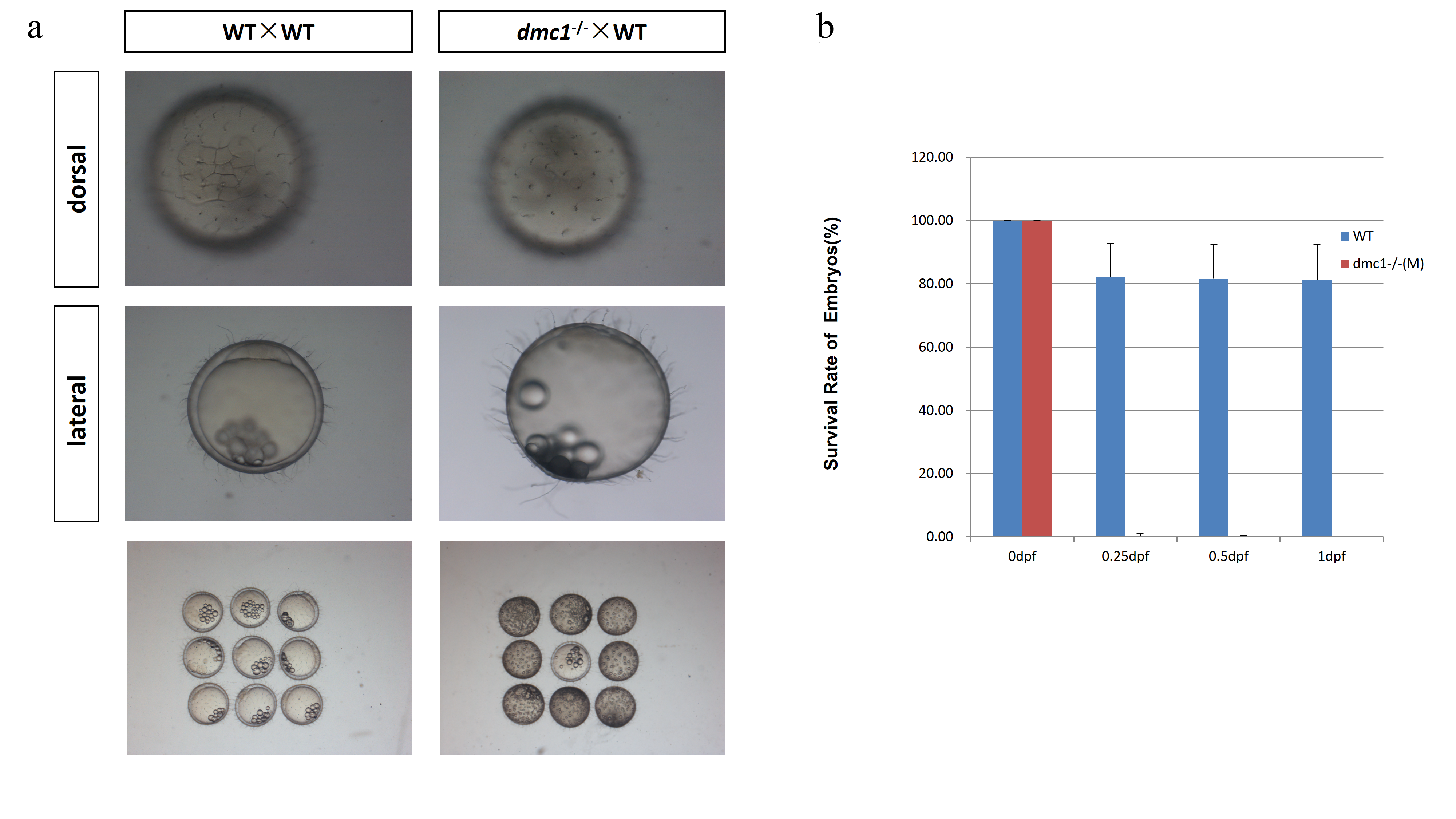
**Supplementary** **Figure S4** Results of the test cross of *dmc1*^−/−^ male medaka with female wild type specimens. (a) The hybrid embryos did not develop normally. (b) All of the hybrid embryos died within one day.

**
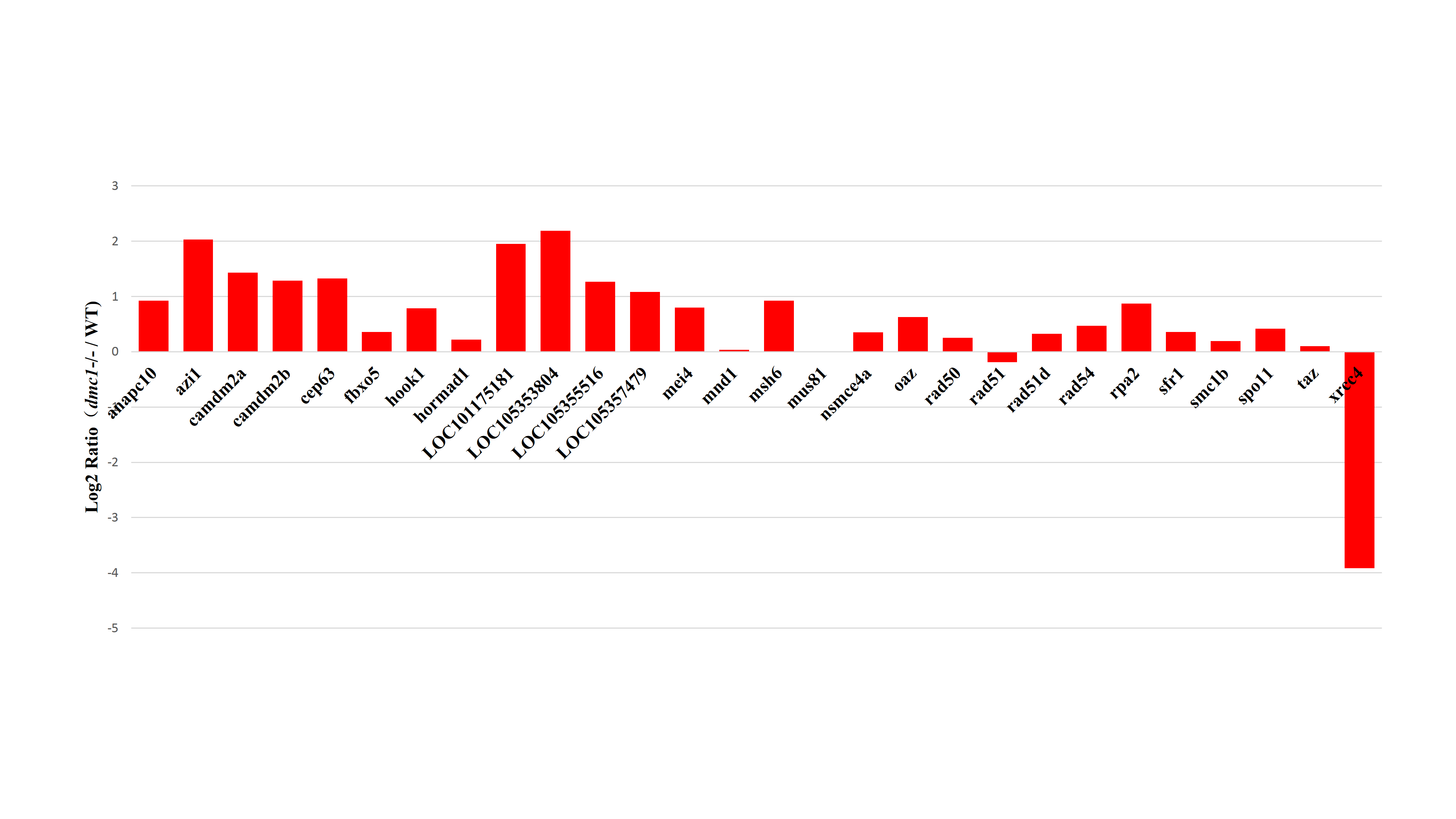
Supplementary** **Figure S5** Quantitative analyses of gene expression levels in wild type and the *dmc1* mutants.

**Supplementary Movie 1** Vitality measurements of the wild type (left) and *dmc1*^−/−^ (right) sperm.

**Supplementary Movie 2** Mobility of the wild type (left) and *dmc1*^−/−^ (right) sperm.
